# Change of School in Early Adolescence and Adverse Obesity-Related Dietary Behavior: A Longitudinal Cohort Study, Victoria, Australia, 2013–2014

**DOI:** 10.5888/pcd12.150042

**Published:** 2015-09-10

**Authors:** Jennifer Marks, Lisa M. Barnett, Steven Allender

**Affiliations:** Author Affiliations: Lisa M. Barnett, School of Health and Social Development, Deakin University, Burwood, Australia; Steven Allender, WHO Collaborating Centre for Obesity Prevention, Deakin University, Geelong, Australia, and School of Health and Social Development, Deakin University, Burwood, Australia.

## Abstract

**Introduction:**

Environments that facilitate energy-dense, nutrient-poor diets are associated with childhood obesity. We examined the effect of a change of school environment on the prevalence of obesity and related dietary behavior in early adolescence.

**Methods:**

Fifteen schools in Victoria, Australia, were recruited at random from the bottom 2 strata of a 5-level socioeconomic scale. In 9 schools, students in grade 6 primary school transitioned to different schools for grade 7 secondary school, whereas in 6 schools, students remained in the same school from grade 6 to grade 7. Time 1 measures were collected from students (N = 245) in grade 6 (aged 11–13 y). Time 2 data were collected from 243 (99%) of the original cohort in grade 7. Data collected were dietary recall self-reported by students via questionnaire, measured height and weight of students, and aspects of the school food environment via school staff survey. Comparative and mixed model regression analyses were conducted.

**Results:**

Of 243 students, 63% (n = 152) changed schools from time 1 to time 2, with no significant difference in weight status. Students who changed schools reported an increase in purchases of after-school snack food, greater sweetened beverage intake, fewer fruit-and-vegetable classroom breaks, and less encouragement for healthy eating compared with students who remained in the same school. School staff surveys showed that more primary than secondary schools had written healthy canteen policies and fewer days of canteen or food services operation.

**Conclusion:**

A change of school environment has negative effects on children’s obesity-related dietary behavior. Consistent policy is needed across school types to support healthy eating in school environments.

## Introduction

Environments that facilitate access to energy-dense, nutrient-poor diets are major contributors to childhood overweight and obesity (1–3). Children living in socioeconomically disadvantaged areas are at greater risk of having poor diets, such as diets high in non-core foods (energy-dense low-nutrient foods such as sweets and snacks) and sugar-sweetened beverages and low in fruits and vegetables, because of differences in nutrition knowledge and food availability and accessibility (4). The school environment has a major influence on health and well-being during childhood and adolescence (5); school-specific (6) and system-wide interventions (7) are needed to promote healthy weight and long-term health benefits (1). Most school-based interventions target either the primary school or the secondary school (8), each associated with its own food environment, including type of food services, meal programs, canteen operations, food options, and pricing policies (9). Despite differences by school type, such as greater availability of snack foods and sugar-sweetened beverages in secondary schools (6), little is known empirically of the effect on eating behavior for students who change school during the transition from primary school to secondary school.

The transition from childhood to adolescence is recognized as a period in which healthy eating behaviors decline (10–12). Knowledge of how a change of school environment affects dietary behavior can help to inform healthy eating and healthy weight interventions in adolescence. The objective of this study was to longitudinally assess whether the prevalence of obesity and obesity-related dietary behavior in early adolescence is affected by a change from primary school to secondary school. A second aim was to compare school food environments by school type. We hypothesized a decline in healthy eating for students who changed schools. We further hypothesized differences in food environments between primary schools and secondary schools that may contribute to changes in dietary behavior.

## Methods

### Design and sample

This was a longitudinal study that followed a cohort of students in grade 6, their last year of primary school (aged 11–13 y) into grade 7, their first year of secondary school, with a change of location and type of school as the exposure of interest. Students were recruited across 2 types of schools (primary and secondary) and 2 types of school transitions. The first type of transition is described by a cohort of children who changed from a discrete primary school in grade 6 to a discrete secondary school in grade 7. The second type of transition is described by a comparison cohort of children who attended the same combined primary–secondary school (grades primary through 9 or primary through 12) for grade 6 and grade 7; henceforth, these schools will be called a P–12 school.

Ethics clearance was obtained from the Deakin University human research ethics committee, and permission to approach Victorian government schools was received from the school state authority. The sample and recruiting strategy is described elsewhere (13). In summary, grade 6 children were recruited from 9 primary and 6 P–12 state government (14) schools, randomly selected from the bottom 2 strata of a 5-level indexed socioeconomic scale (15). All grade 6 students at consenting schools were invited to participate; informed written parental consent for each student was required. For data collection in grade 7, an additional 31 secondary schools (where students who changed schools enrolled) were recruited. School staff members (school principal, canteen manager, and 3 teachers at each school) were invited to participate in a survey on the school food environment.

Parental consent was obtained for 40% (247/623) of invited students. The first data collection phase (time 1) was conducted from October through December 2013 with 245 participating students in their final term of primary school; 2 students were not available. Data collection was repeated (time 2) with 243 participants from time 1 (99% retention rate) 5 to 8 months later from April to June 2014, when participants were in term 2 of secondary school (the school year commences in late January with term 1).

### Anthropometrics

On the day of the survey at both time 1 and time 2, height and weight were measured by trained researchers using a calibrated Charder HM200P height stadiometer (Charder Electronic Co, Ltd.) and a UC-321 weight scale (A&D Australasia Pty, Ltd.). Footwear and heavy outer clothing were removed before measurement according to standardized protocols (16). Height was recorded to the nearest 0.1 cm and weight to the nearest 0.1 kg. All measures were repeated, a third taken if the first 2 measures differed by at least 0.5 cm (height) or 0.5 kg (weight). Average measures were used for analysis. Body mass index (BMI), standardized scores, and weight status were defined using the World Health Organization’s age-specific BMI cut-points (17), a growth standard reference for children aged 5 to 19 years. Statistical calculations were conducted using the WHO reference 2007 module in Stata (StataCorp LP).

### Questionnaires

Students completed behavioral questionnaires using questions from the Eat Well Be Active questionnaire (18), at time 1 and time 2 during a class period. Questions on usual fruit and vegetable intake asked students to indicate their usual quantity during the school day (at recess, lunch, and after school) and their total intake (number of servings) during a full day. Students were also asked to indicate usual school-day intake of 11 non-core food items and 3 sugar-sweetened beverages. The food items were 1) potato chips or a similar snack; 2) chocolate, 3) lollies (candy); 4) muesli or fruit bars; 5) savory biscuits; 6) sweet biscuits; 7) ice cream; 8) hot chips (french fries); 9) pies, pasties, or sausage rolls; 10) hot dogs; and 11) pizza. Students were also asked to specify “other” if applicable. The 3 sugar-sweetened beverages were cordials (a fruit-flavored nonalcoholic drink), fruit juices or drinks, and regular soft drinks. Each intake of an item was scored as 1. Non-core food items represent discretionary foods outside of the 5 main food groups (19) containing saturated fat, added salt, or sugars. In addition, we asked students how often they consumed potato chips, chocolate, lollies, or hot chips on a Likert scale of 1 to 5 (from 1 = don’t eat to 5 = >5 servings). A total non-core food score was calculated by summing scores for usual school-day intake items and daily derived Likert scores per item, according to the survey design (18). Similarly, a total score for sugar-sweetened beverages was derived by summing 1) usual school-day intake scores for each of the 3 sugar-sweetened beverages and 2) daily Likert-scale values for consumption of fruit juice and sugar-sweetened soft drinks (18). An additional non-core food question asked students how often they usually purchased snack food from a shop after school on a scale of 1 (never/rarely) to 5 (every day). The student survey included 4 Likert-scale questions on perceptions of the school environment: 1) “How much does your school encourage students to make healthy eating food choices?” (4-point scale from 1 [a lot] to 4 [not at all]); 2) rating of teachers as healthy-eating role models (5-point scale from 1 [very good] to 5 [very poor]); 3) rating of food and beverage choices available at the school canteen on a 3-point scale from 1 (mostly healthy) to 3 (mostly unhealthy); and 4) frequency of classroom fruit and vegetable breaks on a 5-point scale from 1 (never) to 5 (every day). School perception responses for questions 1, 2, and 3 were reverse-scored for analysis. 

A survey on school environment, designed to assess schools as a setting for promoting healthy eating (20), with separate sections for school principals, teachers, and canteen managers, was completed by school staff. School principal surveys comprised 8 questions, including questions on school proximity to external food outlets, food policies, and food service operations. School teacher surveys comprised 8 questions on food policies and promotion of healthy eating. Teacher responses per school were averaged to 1 score per variable for each school. School canteen manager surveys comprised 11 food service questions, including questions on frequency of operation and types of foods provided. 

### Statistical analyses

A minimum sample size of 120 students (60 who changed schools, 60 who did not change schools) was needed to achieve 80% power for detecting a change in student behavior indicated by the 5-point Likert scale variables. To check for independence of schools before analysis, intraclass correlations were determined by student age, sex, and weight status and by each dependent variable at baseline to examine any school clustering effect. Proportions and means were calculated for student demographic variables with comparisons between students who changed schools and student who did not change schools. Mean values were calculated for student dietary behaviors and school food environment perceptions for both time 1 and time 2. Changes in weight status and dietary behavior from time 1 to time 2 and differences in student perceptions of school food environments between school types were assessed by using the exact McNemar test or the Bowker paired test of proportions for categorical variables or a paired *t* test of means for continuous variables. To explore the differential effect on behavior by the binary outcomes for weight status (underweight or healthy weight vs overweight or obese) and between changing schools or not changing schools, mixed model regression analyses for longitudinal data were conducted from time 1 to time 2 after adjusting for age and sex in change-of-school models and individual scores at baseline in all models. To adjust for school clustering, hierarchical models (level 1, individual; level 2, school) were fitted for a behavior where the intraclass correlation was greater than 0.20. This was applicable only for the variable “fruit and vegetable classroom break” (intraclass correlation = 0.43). Linear regression was used to model continuous variables, and Poisson regression (generating incidence rate ratios) was used to model count-dependent variables. No values were assumed or imputed for missing values. Data on health ratings of choices in canteens collected from 39 (16%) students at time 2 were excluded from analysis because of students not having an available canteen at time 1 to make a paired comparison. No significant difference in responses at time 2 were found between students who had a canteen option at time 1 and students who did not have a canteen option at time 1 (χ^2^ = 2.04; *P* = .36). Because of variations in staff response rates, statistical analyses of the school food environment were not conducted. Descriptive comparisons by school type were made instead to provide context of student exposure to different food environments. Significance was set at *P* < .05 (2-sided). All analyses were conducted using Stata 12.0 software (StataCorp LP).

## Results

Of 243 students (98 boys, 145 girls) participating at both time points, 152 (63%) changed schools; 91 (37%) remained at the same P–12 school. At time 1, mean age was 12.2 years (12.3 y, boys; 12.2 y, girls; *P* = .003); at time 2, 12.7 years. No significant differences by ethnicity were found between boys and girls. Most (85%) were born in Australia. We found no significant differences for age, sex, or weight status by individual school at baseline, by school type at time 1 (between primary and P–12 schools), or by school type at time 2 (between secondary and P–12 schools).

Complete anthropometric data were collected for 238 of 243 (98%) participants at time 1 and time 2. Average prevalence of overweight/obesity was 35% in grade 6 (33% of students intending to change school, 39% of students not intending to change school; *P* = .42). In grade 7, average prevalence of overweight/obesity was 37% (34% of students who changed school, 42% of students who did not change school; *P* = .23).

The daily intake of non-core food items (−1.2; 95% CI, −1.7 to −0.7; *P* < .001) and sugar-sweetened beverages (−0.3; 95% CI, −0.5 to −0.1; *P* < .001) decreased after the transition to secondary school (Table 1). During the same period, the usual intake of school-day fruit (−0.2; 95% CI, −0.4 to −0.1; *P* = .003) and school-day vegetables (−0.2; 95% CI, −0.3 to −0.1; *P* < .001) also decreased, and perceptions of the school healthy eating environment declined. 

**Table 1:** Change in Student Dietary Behavior and School Perceptions From Time 1 (Grade 6) to Time 2 (Grade 7), Victoria, Australia, 2013–2014

Variable (Potential Range for Each Variable)	No. of Responses	Time 1: Grade 6, Mean (SD) [95% CI]	Time 2: Grade 7, Mean (SD) [95% CI]	Time 1 to Time 2 Difference	
				Mean (SD) [95% CI]	*P* a
Score for daily intake of non-core food (range, 0–33)	242	5.0 (4.2) [4.4 to 5.5]	3.8 (2.3) [3.5 to 4.1]	−1.2 (4.0) [−1.7 to −0.7]	<.001
Score for daily intake of sugar-sweetened beverages (range, 0–11)	235	2.0 (1.5) [1.8 to 2.2]	1.7 (1.2) [1.5 to 1.8]	−0.3 (1.5) [−0.5 to −0.1]	<.001
Usual daily frequency of fruit consumption (5-point scale of 1 = don’t eat to 5 = >5 servings)	242	3.2 (0.8) [3.1 to 3.3]	3.1 (0.8) [3.0 to 3.2]	−0.1 (1.2) [−0.2 to 0]	.20
Usual school-day (recess, lunch, after school) fruit consumption (range, 0–6)	242	1.2 (1.1) [1.0 to 1.3]	0.9 (0.9) [0.8 to 1.1]	−0.2 (1.2) [−0.4 to −0.1]	.003
Usual daily frequency of vegetable consumption (5-point scale of 1 = don’t eat to 5 = >5 servings)	243	3.2 (0.8) [3.1 to 3.4]	3.2 (0.8) [3.1 to 3.3]	−0.1 (0.8) [−0.2 to 0]	.22
Usual school-day (recess, lunch, after school) vegetable consumption (range, 0–3)	243	0.4 (0.7) [0.3 to 0.5]	0.3 (0.5) [0.2 to 0.3]	−0.2 (0.9) [−0.3 to −0.1]	<.001
Frequency of fruit/vegetable classroom breaks (5-point scale from 1 = never to 5 = every day)	242	3.0 (1.8) [2.8 to 3.2]	1.5 (1.1) [1.4 to 1.6]	−1.5 (2.0) [−1.8 to −1.3]	<.001
Rating of canteen choices (3-point scale from 1= mostly unhealthy to 3 = mostly healthy)	204	1.7 (0.6) [1.7 to 1.8]	1.7 (0.6) [1.6 to 1.8]	−0.1 (0.7) [−0.2 to 0]	.14
School encourages healthy eating choices (4-point scale from 1 = not at all to 4 = a lot)	240	2.1 (0.9) [2.0 to 2.3]	1.7 (0.9) [1.6 to 1.9]	−0.4 (1.1) [−0.5 to −0.3]	<.001
Teachers as healthy eating role models (5-point scale from 1 = very poor to 5 = very good)	241	3.8 (1.0) [3.7 to 4.0]	3.6 (0.9) [3.5 to 3.8]	−0.2 (1.1) [−0.3 to −0.1]	.006
Frequency of buying snack food from shop after school (5-point scale from 1 = never to 5 = every day)	243	1.9 (1.0) [1.8 to 2.1]	1.9 (1.0) [1.8 to 2.1]	0 (1.1) [−0.1 to 0.2]	.86

Abbreviations: CI, confidence interval; SD, standard deviation.

a Determined by using paired *t* test of means for difference between time 1 and time 2.

School environment surveys were received from 45% (n = 20) of school principals, 2 from six P–12 schools, 7 from 9 primary schools, and 11 from 31 secondary schools (Table 2). Returned surveys from 48% (66 of 138) of school teachers represented 72% (33 of 46) of participating schools, comprising 100% (6 of 6) of P–12 schools, 89% (8 of 9) primary schools, and 61% (19 of 31) of secondary schools. Canteen manager surveys were received from 33% (13 of 39) schools with canteen or food services, representing 33% (2 of 6) of P–12 schools, 50% (2 of 4) primary schools, and 31% (9 of 29) of secondary schools. 

**Table 2:** School Food and Drink Environment by Type of School and Type of Staff Member Responding to Questionnaire^a^, Victoria, Australia, 2013–2014

Type of Administrator/Question	Combined Primary and Secondary Schools (n = 6)	Primary School (n = 9)	Secondary School (n = 31)
**School principals**			
No. of principal responses	2	7	11
Proximity of nearest milk bar^b^/fast food outlet (4-point scale from 1 [≤ 100 m] to 4 [>1 km]), mean	3.0	2.4	2.7
Food service operating at the school in the last 12 months, % yes	100	86	91
Food service operated by external food company, % yes	0	33	64
Food service an important source of school funds, % yes	0	0	20
Food service exclusive contract with soft drink/other foods, % yes	0	0	25
Written food policy promoting nutrition and healthy eating, % yes	0	71	40
Students allowed to drink water in the classroom during class-time, % yes	100	100	100
School has a school vegetable garden, % yes	50	86	70
**School teachers^c^**			
No. of schools represented by teacher responses	6	8	19
No. of teacher responses	11	16	39
Existence of written school nutrition or healthy canteen policy (0 = no; 1 = yes), mean	0.3	0.7	0.2
School canteen provides foods high in nutritional value (1 = strongly disagree to 5 = strongly agree), mean	2.5	3.5	2.8
Proportion of teachers are aware of nutrition or healthy canteen policy (1 = very few to 5 = all), mean	2.4	4.1	2.5
Proportion of parents aware of nutrition or healthy canteen policy (1 = very few to 5 = all), mean	1.9	3.3	2.4
Nutrition or healthy canteen policy compliance in last 12 months (1 = very poor to 5 = very good), mean	2.8	4.4	3.6
Parental support for healthy eating in last 12 months (1 = very low to 5 = very high), mean	3.0	2.9	2.7
Proportion of teachers as good healthy eating role models (1 = very few to 5 = all), mean	4.5	4.3	3.5
Effectiveness of promoting healthy eating among students (1 = not effective to 4 = very effective), mean	2.8	2.9	2.7
**Canteen managers^d^**			
No. of canteen manager responses	2	2	9
No. of days per week school food service operated, mean	4.0	2.0	5.0
School food service open to students at recess, % yes	100	50	100
School food service open to students at lunch time, % yes	50	100	100
Fruit usually available from school food service, % yes	50	100	100
Vegetables/salad usually available from school food service, % yes	100	50	100
Lollies/confectionary/chocolate usually available from school food service, % yes	0	0	33
Pies/sausage rolls/hot chips usually available from school food service, % yes	50	50	100
Crisps/chips usually available from school food service, % yes	50	0	89
Sugar-sweetened drinks usually available from school food service, % yes	50	0	56
Pricing policy to encourage sale of healthy foods at reduced cost, % yes	100	50	56
Food service routinely promotes healthy food choices, % yes	100	50	100

a School staff members (school principal, canteen manager, and 3 teachers at each school) were invited to participate in a survey on the school food environment.

b Small truck stop, corner store, or convenience store.

c Teacher responses per school were averaged to 1 score per variable for each school.

d Thirty-nine schools had canteen or food services: 6 P–12 schools, 4 primary schools, and 29 secondary schools.

Secondary school food environments were generally perceived as less conducive to promoting healthy eating than primary or P–12 schools (Table 2). Fewer secondary schools had healthy eating policies; they also had lower levels of compliance and awareness of their existence. Fewer teachers in secondary schools than in primary or P–12 schools perceived themselves as healthy eating role models. Among primary and secondary schools that operated a canteen or food service, no significant differences in type or pricing of foods by school type were found. All secondary school canteens operated each school day; primary schools averaged 2 days of operation per week.

Mixed model regression analysis (Table 3) identified more pronounced negative changes in some behaviors and perceptions among students who changed school than among students who remained in the same school. Participants who changed schools had a significantly smaller reduction in the mean score (−0.2) for sugar-sweetened beverage intake than students who did not change schools (−0.6) (*P* = .03 for difference). A change of schools was also associated with a decline in the frequency of fruit and vegetable classroom breaks (mean difference, −0.6; *P* = .01) and a reduction in school encouragement to eat healthily (mean difference −0.3; *P* = .03). Frequency of purchasing snack foods after school increased among students who changed schools and decreased among students who remained in the same school (mean difference 0.3; *P* = .03). No significant associations were found between weight status and dietary behavior.

**Table 3:** Effects on Student Dietary Behavior and School Perceptions From Time 1 (Grade 6) to Time 2 (Grade 7) by Change or No Change of School and Weight Status, Victoria, Australia, 2013–2014

Dependent Variable	Change of School		Change of School vs No Change of School^a^		Overweight/Obese vs Not Overweight	
	Yes	No	Difference (95% CI)	*P* b	Difference (95% CI)	*P* b
**Continuous**						
Score for daily intake of non-core food (range, 0–33)	−1.0	−1.6	0.6 (−0.4 to 1.7)	.25	−0.6 (−1.7 to 0.5)	.26
Score for daily intake of sweetened beverages (range, 0–11)	−0.2	−0.6	0.4 (0 to 0.8)	.03	−0.1 (−0.5 to 0.3)	.48
Usual daily frequency of fruit consumption (5-point scale of 1 = don’t eat to 5 = >5 servings)	−0.1	0	0 (−0.3 to 0.2)	.67	−0.1 (−0.4 to 0.1)	.20
Usual daily frequency of vegetable consumption (5-point scale of 1 = don’t eat to 5 = >5 servings)	−0.1	0	−0.1 (−0.3 to 0.1)	.41	−0.1 (−0.3 to 0.1)	.41
Frequency of fruit and vegetable classroom breaks (5-point scale from 1 = never to 5 = every day)^c^	−1.7	−1.1	−0.6 (−1.1 to 0.1)	.01	−0.2 (−0.7 to 0.3)	.38
Rating of canteen choices (3-point scale from 1= mostly unhealthy to 3 = mostly healthy)	−0.1	0	0 (−0.2 to 0.2)	.75	0.2 (0 to 0.4)	.08
School encourages healthy eating choices (4-point scale from 1 = not at all to 4 = a lot)	−0.5	−0.2	−0.3 (−0.6 to 0)	.03	−0.2 (−0.5 to 0.1)	.21
Teachers as healthy eating role models (5-point scale from 1 = very poor to 5 = very good)	−0.2	−0.2	0 (−0.3 to 0.3)	.83	0 (−0.3 to 0.3)	.89
Frequency of buying snack food from shop after school (5-point scale from 1 = never to 5 = every day)	0.1	−0.2	0.3 (0 to 0.6)	.03	−0.1 (−0.4 to 0.2)	.71
**Categorical**	—	—	IRR (95% CI)	*P* d	IRR (95% CI)	*P* d
Usual school-day (recess, lunch, after school) fruit consumption (range, 0–6)	—	—	0.9 (0.6–1.2)	.38	1.0 (0.7–1.4)	.89
Usual school-day (recess, lunch, after school) vegetable consumption (range, 0–3)	—	—	1.2 (0.6–2.2)	.59	0.8 (0.4–1.4)	.40

Abbreviation: —, does not apply; CI, confidence interval, IRR, incidence rate ratio.

a Change of school models adjusted for age and sex.

b *P* value for test value of linear regression interaction effects.

c Multilevel models account for clustering at the school level.

d *P* value for test value of Poisson regression interaction effects.

## Discussion

This study is the first to examine the effect of a change of school environment in early adolescence on obesity and obesity-related dietary behavior. Although no significant effect on BMI was found, obesity prevalence was high across the school transition. Overall, we found both positive and negative behavioral changes in all students transitioning from grade 6 to grade 7. We also found that participants who changed schools when transitioning from primary school to secondary school, compared with students who did not change schools, were exposed to less healthy eating environments and had poorer dietary behaviors.

Contrary to existing literature (10–12), intake of non-core food items and sugar-sweetened beverages decreased on average for all students during the study period. This decrease is not explained by seasonal influence (spring-summer [October–December] to autumn [April–June]); students who changed schools had less of a decline in intake of non-core food items and sugar-sweetened beverages, where a seasonal influence would be expected to affect the diet and intake of all students similarly. A Canadian study found a positive association between inadequate guidelines on healthy eating and the availability and intake of sugar-sweetened beverages at school (21). In contrast, an Australian study found that adolescent attitudes, peer modeling, and intentions were stronger predictors than the physical school environment of the consumption of sugar-sweetened beverages and snack foods (22). Our findings suggest the change of school environment negatively affected eating behaviors. Further evidence is needed to determine the relative contribution of the change in school physical or school social environment on dietary behavior.

In line with trends among adolescents (23), school-day fruit and vegetable intake decreased overall for all students. Despite a drop overall in the frequency of fruit and vegetable classroom breaks from primary to secondary school, with a greater decrease in frequency for students who changed schools, a change of school did not significantly change level of intake. A recent systematic review supports these results, finding that individual preference and parental intake were more influential than the school food environment on fruit and vegetable intake among children and adolescents of low socioeconomic status (24). These findings may reflect already inadequate fruit and vegetable intake among adolescents (4,10–12), with the minimal amount consumed during the school day not having a significant impact on overall intake. To increase fruit and vegetable intake at school, efforts must take into account external influences as well as a review of the school food environment.

Local food environments in and around secondary schools can influence dietary intake among adolescents (6,25,26). Data on the proximity of the nearest food outlet did not explain an increase in snack food purchases by students who changed their school environment. A decline in healthy eating behavior among secondary school students might be explained by a combination of school factors, including lack of compliance or poor compliance with healthy food policies in canteens that operate daily, student ratings of teachers as poorer role models of healthy eating, teacher perceptions of a lower proportion of teachers acting as healthy role models, and a larger decline in the frequency of fruit and vegetable classroom breaks at secondary schools than at primary schools. In Australia, national guidelines for implementing healthy canteens in schools are available, but conforming to guidelines is not mandatory (27), possibly explaining the differences in policy implementation and adherence by school type. Interventions that improve the school food environment can have positive effects on weight status, food choices, and eating behavior (28,29). Our findings also suggest that effective policies and interventions need to be implemented and practiced consistently by all types of schools as children move from one educational institution to another.

A major strength of this study was the high retention rate of longitudinal cohort participants, minimizing any potential bias from loss to follow-up. A further strength was the study design, which incorporated randomized and independent primary schools and P–12 schools at baseline. The study also had limitations. Pubertal status, a potential confounder in studies of obesity (30), was not known and hence was not adjusted for in the analysis. A further limitation was the use of student self-report for the data on dietary recall, which has the potential for underestimation and overestimation. However, validated instruments were used to analyze behavioral differences. Another limitation was the low staff response rates for the school environment surveys, preventing an analysis of associations between dietary behavior and environment. However, staff responses aligned with existing evidence, providing context to the different school environments to which students were exposed. Because of the study design by type of school and geographic location, results may not be generalizable to all school systems for students in early adolescence.

This study demonstrates that a change of school environment can negatively affect dietary behaviors that put adolescents at risk of obesity. Our findings have implications for policy, particularly policies on healthy eating and canteen operation in all levels of schooling. The disruptive effect on eating behavior of the transition from a primary school to a secondary school demonstrates the need for consistent messages to create environments that support healthy eating for the prevention of obesity and promotion of long-term health.
